# Aging in multiple sclerosis: from childhood to old age, etiopathogenesis, and unmet needs: a narrative review

**DOI:** 10.3389/fneur.2023.1207617

**Published:** 2023-06-02

**Authors:** Nicola Capasso, Eleonora Virgilio, Antonio Covelli, Beatrice Giovannini, Matteo Foschi, Federico Montini, Martina Nasello, Annacarmen Nilo, Elio Prestipino, Giuseppe Schirò, Silvia Sperandei, Marinella Clerico, Roberta Lanzillo

**Affiliations:** ^1^Department of Neuroscience, Reproductive Sciences and Odontostomatology, Federico II University of Naples, Naples, Italy; ^2^Multiple Sclerosis Unit, Policlinico Federico II University Hospital, Naples, Italy; ^3^Neurology Unit, Department of Translational Medicine, AOU Maggiore della Carità Novara, University of Eastern Piedmont, Novara, Italy; ^4^Department of Neurology, Santi Antonio e Biagio e Cesare Arrigo Hospital, Alessandria, Italy; ^5^Neurology Unit, Department of Clinical and Experimental Medicine, University of Pisa, Pisa, Italy; ^6^Department of Neuroscience, MS Center, S. Maria delle Croci Hospital, AUSL Romagna, Ravenna, Italy; ^7^Department of Biotechnological and Applied Clinical Sciences (DISCAB), University of L’Aquila, L’Aquila, Italy; ^8^Neurology Unit, IRCCS San Raffaele Scientific Institute, Milan, Italy; ^9^Neurology Unit, Department of Neurosciences, Mental Health and Sensory organs (NESMOS), Sapienza University of Rome, Rome, Italy; ^10^Clinical Neurology Unit, Department of Head, Neck and Neurosciences, Santa Maria della Misericordia University Hospital, Udine, Italy; ^11^UOSC Neuro-Stroke Unit, AORN Antonio Cardarelli, Naples, Italy; ^12^Section of Neurology, Department of Biomedicine, Neurosciences and Advanced Diagnostics (BiND), University of Palermo, Palermo, Italy; ^13^Section of Neurology, Department of Medicine and Surgery, University of Perugia, Perugia, Italy; ^14^Department of Clinical and Biological Sciences, University of Turin, Turin, Italy

**Keywords:** multiple sclerosis, aging, pediatric-onset, late-onset, immunosenescence, risk factors, unmet need, engagement

## Abstract

Multiple sclerosis (MS) primarily affects adult females. However, in the last decades, rising incidence and prevalence have been observed for demographic extremes, such as pediatric-onset MS (POMS; occurring before 18 years of age) and late-onset MS (corresponding to an onset above 50 years). These categories show peculiar clinical-pathogenetic characteristics, aging processes and disease courses, therapeutic options, and unmet needs. Nonetheless, several open questions are still pending. POMS patients display an important contribution of multiple genetic and environmental factors such as EBV, while in LOMS, hormonal changes and pollution may represent disease triggers. In both categories, immunosenescence emerges as a pathogenic driver of the disease, particularly for LOMS. In both populations, patient and caregiver engagement are essential from the diagnosis communication to early treatment of disease-modifying therapy (DMTs), which in the elderly population appears more complex and less proven in terms of efficacy and safety. Digital technologies (e.g., exergames and e-training) have recently emerged with promising results, particularly in treating and following motor and cognitive deficits. However, this offer seems more feasible for POMS, being LOMS less familiar with digital technology. In this narrative review, we discuss how the aging process influences the pathogenesis, disease course, and therapeutic options of both POMS and LOMS. Finally, we evaluate the impact of new digital communication tools, which greatly interest the current and future management of POMS and LOMS patients.

## 1. Introduction

Multiple Sclerosis (MS) is a chronic, inflammatory, immune-mediated disease of the central nervous system disease (106). MS is one of the most relevant causes of neurological disability in young people, with an important social and economic impact (38). An early diagnosis is crucial for managing MS evolution and reducing morbidity and long-term effects (103). MS has a multifactorial etiology; young females are the most affected population (with a peak incidence between 20 and 40 years old). However, MS can emerge in all age groups, including pediatric patients (pediatric-onset MS, POMS), corresponding to children before 18 years of age (2–10% of total cases), and after 50 years of age (late-onset MS, LOMS; with a prevalence ranging from 1.1–21.3% of cases depending from cut-offs and diagnostic methods considered) (82). These demographic extremes present different clinical and pathogenetic characteristics (71).

The clinical phenotype of POMS differs from adult patients. POMS patients generally experience a more aggressive disease onset with disabling clinical symptoms, a polyfocal presentation at disease onset, and a higher relapse rate early in the disease course (60). In recent decades, evidence confirmed that early axonal damage in MS patients contributes to clinical disability and progression from early disease stages (113). In POMS, acute axonal injury following inflammatory demyelinating lesions is more pronounced than in the adult counterparts (89). In contrast, LOMS patients are more likely to convert in secondary progressive phases, suggesting that they may experience a more evident chronic axonal loss associated with physiological aging (1, 111). Children are less likely to develop primary or secondary progressive MS, and 98% of POMS present with a relapsing–remitting (RR) course, compared with 84% of adult patients and 50% of LOMS (3).

POMS and LOMS are also challenging during the diagnostic workup. For POMS, it is essential to rule out other disorders that may mimic MS and demyelinating syndromes that can occur more likely than MS in childhood, such as mog-associated disease (MOGAD) and acute disseminated encephalomyelitis (ADEM) (10). POMS must not only be differentiated from acute ADEM or MOGAD, but there is also an extensive list of other disorders that can mimic MS, which need to be excluded. Such diseases include neuromyelitis optica spectrum disorder (NMOSD), systemic lupus erythematosus (SLE), neurosarcoidosis, Sjögren syndrome, leukodystrophies, hereditary metabolic diseases, and encephalitic or meningoencephalitis infectious etiologies. Inflammation of the brain during critical developmental periods, including myelinogenesis in adolescence, may irreparably damage neural networks involved in cognition. This damage may also lead to the reduced brain and deep gray matter volumes in adulthood reported in POMS relative to sex- and age-matched patients with AOMS independent of disease duration (73). Several studies have demonstrated that individuals with POMS have slower disease progression than their adult-onset counterparts, particularly during the early stages of the disease. This discrepancy may suggest greater plasticity, less neurodegeneration, and potentially more repair and remyelination in the younger nervous system (4).

In the last decades, the average age of MS patients has progressively increased, as well as the number of patients with LOMS or very late onset MS (VLOMS, onset after 60 years of age) (13, 71, 102, 112). The yearly incidence of LOMS and VLOMS represents 3.4–4.8 and 0.5% of all new diagnoses (50, 91, 112). Moreover, an increasing number of young MS patients are getting older, along with the general population trend (71, 112). Better diagnostic accuracy, longer life expectancy, and the introduction of specific disease-modifying treatments (DMTs) are among the factors leading to the growing number of older MS patients (112).

The management of older MS patients represents a clinical and therapeutic challenge, and the risk of misdiagnosis is higher than in younger patients. This is mainly due to the higher prevalence of comorbidities and immunosenescence. Indeed, the clinical onset of older MS patients is generally characterized by motor symptoms (101) potentially sharing similar features with deficits from other neurological disorders (i.e., cerebrovascular diseases) prevalent in older age. In addition, LOMS patients are more frequently male and tend to have a progressive form of the disease (8, 68). Moreover, long disease duration is associated with a worse prognosis in old MS patients (85). Some radiological and laboratory biomarkers such as spine involvement (usually spared in vascular diseases), the presence of lesions in the septum callosum (typical of MS), and the presence of oligoclonal bands in cerebrospinal fluid could be helpful to support MS diagnosis in older patients (20).

Finally, demographic extremes, POMS and LOMS, have different clinical, pathogenic, and prognostic characteristics, with 16% of LOMS reaching a score of 6 in the Expanded Disability Status Scale (EDSS), compared to the 15% of POMS (82). Thus, the clinical management, therapeutic approach, and social engagement of these two groups of patients are completely different.

Here we present a narrative review discussing how the aging process influences the pathogenesis, disease course, and therapeutic options of both POMS and LOMS. Finally, we evaluate the impact of new digital communication tools, which greatly interest the current and future management of POMS and LOMS patients.

## 2. Pediatric-onset multiple sclerosis patients

### 2.1. Genetic background

**Figure 1:**
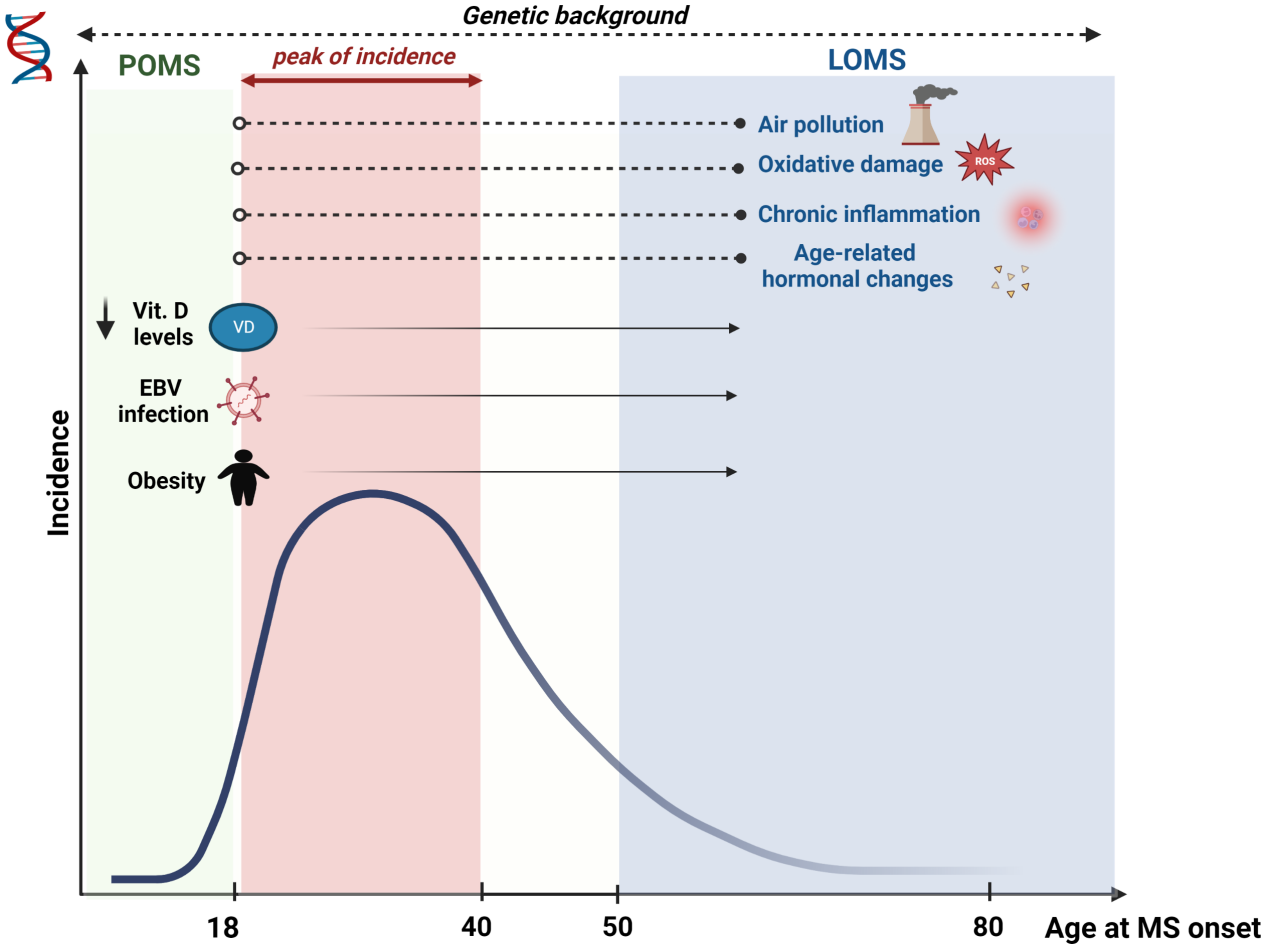
Multiple Sclerosis (MS) incidence and effects of risk factors according to the age of onset. MS incidence gradually increases from childhood, peaking between the second and the fourth decade. Subsequently, a gradual decrease is seen with aging. Several risk factors have been associated mainly with POMS (MS onset <18 years), although their effect persists even in older ages (i.e., obesity, low blood vitamin D levels, viral infections, especially EBV). Other risk factors have been mainly associated with LOMS (MS onset >50 years), including air pollution, oxidative damage, chronic inflammation and hormonal changes. Although impacting on MS risk since childhood, the exposure to these factors may imply a longer time to increase the risk of developing MS. Lastly, genetic background influences MS risk throughout the lifetime.

Several pieces of evidence support the contribution of genetic factors in the onset of POMS (Figure 1). Moreover, some of the variants identified as possible genetic risk factors increase the susceptibility to the onset of the disease during the pediatric age and to the onset in adulthood, suggesting that the two forms of MS share similar and superimposable biological processes (42). Human leukocyte antigen (HLA) genetic variants and non-HLA variants extend the risk of developing MS in childhood (42). However, mainly the HLA molecules, among these, those of class II, confer a greater genetic susceptibility. Not surprisingly, the polymorphisms of the classic risk factor for adult MS HLA–DRB1*15:01 are also associated with an increased risk of developing the disease in childhood, although a greater association is described for adult-onset MS (7). The HLA-DRB1*03 allele was also identified - for the first time in a population of pediatric MS patients of Greek origin - as a genetic risk factor compared to healthy controls and adult MS patients. In particular, its presence identifies patients with greater inflammatory disease activity and more relapses, mainly with the involvement of the thoracic spinal cord (43). The HLA-DP alleles, although less studied than HLA-DR alleles, seem involved in the pathogenetic mechanisms of the disease. In particular, HLA-DPB1*03 allele, already known as allele risk for adult MS, plays a role also for pediatric MS, while the HLA-DPB1*04 allele has shown a protective role for the onset of the disease in both adults and children (6). Furthermore, it is unclear whether the known genetic susceptibility factors for disease onset may also influence the relapse rate. A genotyping analysis by Graves and collaborators on 181 patients from two pediatric MS centers in the United States found no association between the number of relapses and genetic risk score for non-HLA genes. Instead, HLA-DRB1*15 was found to modify the association of vitamin D status with relapse rate (44). It should be noted that many of the biological processes that play a decisive role in the onset of MS, including pediatric MS, result from complex gene–environment interactions. According to this view, the disease emerges from genetic susceptibility under the impulse of one or more environmental exposure factors. For example, the risk of pediatric MS associated with high levels of environmental pollutants in the air appears to be greater in patients carrying the GG genotypic variant of the single nucleotide polymorphism rs928264 (G/A) within CD86 or in patients with HLA polymorphisms DRB1*15:01 (119).

In conclusion, although several genetic variants have been identified as susceptibility factors for the onset of MS, each of these individually plays a marginal role in the development of the disease, and perhaps more genes participate together in increasing the risk of the disease. This would lead to considering both pediatric and adult-onset MS as a polygenic disease. Furthermore, the final expression of the disease is also the result of a complex interaction with environmental factors, whose phases and exact mechanisms are not yet fully known, and any future treatments should also consider this aspect.

### 2.2. Environmental factors

The environmental risk factors involved in the etiopathogenesis of pediatric MS are many, but for some of them, the data collected so far in the literature are few. Childhood obesity has often been proposed as an environmental risk factor in pediatric MS. A high BMI is associated with a higher risk of prepubertal and postpubertal pediatric MS in both boys and girls (26), and with a higher risk of MS onset in both females aged 7–13 years and in males between 8 and 10 years of age (80). A key role in the pathogenesis seems to be linked to increased leptin and pro-inflammatory cytokines (IL-2, IL-6, IFN-γ, and TNF-α) and reduced adiponectin levels in patients compared to controls (54, 84). Furthermore, obesity, through the dysregulation of Th17 and T-reg cell activity, could change the intestinal microbiome favoring the development of a pro-inflammatory environment (108). Furthermore, POMS may be less likely to consume sufficient iron compared to controls (87). Therefore, the promotion of a healthy diet and good weight control allows a modulation of the immune response in the pediatric age, potentially preventing chronic inflammatory diseases such as MS. Breastfeeding seems to constitute a protective factor for the development of pediatric MS in the perinatal period, data suggest a potential reduction of autoimmune dysregulation and an increase in the activity of the immune system against pathogens. In this regard, proposing early breastfeeding, especially to women of gestational age with a family history of MS, would constitute a potential preventive therapy against the development of pediatric MS (46). In adults, low levels of vitamin D, due to a low sun exposure, which in its active form works by inhibiting the activity of inflammatory cells involving both vitamin D and non-vitamin D pathways, may increase susceptibility to MS, a finding not yet confirmed in children, although vitamin D levels are lower than the normal range in most pediatric MS patients (48, 79). Spending more time in the sun during summer may be strongly protective against developing POMS (100). In a previous meta-analysis, it was reported strong evidence for a casual and independent association between low serum concentrations of vitamin D and increased BMI and risk of POMS (41). Recent metagenomic analyses show an altered gut microbiome-related metabolic potential in POMS patients compared to controls, including higher breakdown of lipopolysaccharide molecules, higher prevalence of a methane producing pathway from *Archaea* and depletion of the lactate fermentation pathway, but lower resistant starch metabolism (76, 77). In a recent USA case–control study gut microbiota diversity was similar for POMS and controls, however at the gut-community-network level differences were observed, in particular POMS patients exhibited an overrepresentation of highly connected opportunistic pathogens, suggesting a possible contribute to MS pathogenesis (109).

Very recent works, using data from millions of US military recruits monitored over 20 years, proposes EBV as the leading cause of MS, showing that the risk increases approximately 32-fold after infection with EBV but does not appear to increase after infection with other viruses, even with a transmission mechanism similar to CMV (16). About that, EBV negative to positive seroconversion generally increases with age with a major incidence peak in early childhood and a second peak, especially for females, around puberty, coinciding with the approximate age of mononucleosis and with the highest female prevalence in MS (28, 34, 35, 63, 66, 81, 98). EBV infection is related to pediatric MS, and generally, all children with MS are EBV seropositive, whereas the positivity rate is considerably lower in healthy children (11, 15, 69, 86, 90). French authors in 2006 demonstrated an absence of correlation between an increased risk of the onset of MS after administration of the vaccine against the hepatitis B virus (HBV), nor between an increase in the relapse rate in a patient with a first episode of demyelinating disease of the CNS after HBV vaccination (74). The same authors then speculated on the risk of MS onset in childhood concerning exposure to secondhand smoke, which seems to be doubled compared to patients whose family members do not smoke and to be even higher in case of prolonged exposure aged 10 years or older (75). In the past, an increased susceptibility to MS has been described in patients with type I diabetes mellitus (DM). Data from American case–control studies show a 3- to 10-fold increased risk of developing MS in children of mothers with diabetes mellitus, particularly mothers who have had diabetes during pregnancy (44). Furthermore, a recent cohort study of newborns in Denmark from 1978 to 2008 observed a doubled risk of MS in children of mothers with pre-gestational diabetes compared with children of non-diabetic mothers (83). These data underline the importance of the anamnestic moment as an early opportunity to obtain such information even before conception so as to intercept the possibility of developing pediatric MS in this category of patients as soon as possible. However, it is still unclear today whether a common cause underlies both pathologies. However, there are no studies able to better define the intricate pathway involving OPN, Th17 cells and dendritic cells, which seems to link MS and type I DM.

### 2.3. Treatments for POMS

In POMS, relapse treatment is similar to the adult form of MS, consisting of high-dose steroids. Due to the high relapse rate and the improved recovery from relapses of POMS, early DMTs start is strongly recommended (23, 27). Low-efficacy DMTs available for POMS are interferon-β and glatiramer acetate. Although no randomized trials have been conducted, these drugs have shown adult-like efficacy and tolerance in various retrospective studies, and they have been at the base of treatment in pediatric patients for years (32). In case of failure or poor tolerability, high-efficacy DMTs should be considered. Several oral treatments are available in adult patients: Fingolimod, teriflunomide, and dimethyl fumarate. In US a multicenter study showed that initial treatment of POMS with this newer DMTs led to better disease activity control compared to injectables, supporting a greater effectiveness. However long-term safety data are still lacking (61). In POMS, the randomized trial PARADIGMS demonstrated an 81.9% efficacy of Fingolimod in reducing the annualized recurrence rate compared with IFNs and at least a 53% reduction in the annualized rate of new lesions on MRI (24). Furthermore, the greater efficacy of this drug has been highlighted in children than in adults, probably due to a greater inflammatory component of the juvenile form (29). Teriflunomide has been approved as a treatment in patients from 10 to 17 years old thanks to the data from the phase III, trial, TERIKIDS: the drug was well tolerated and had an excellent safety profile in this population (25). The two trials, FOCUS and CONNECTED, showed similar long-term safety and efficacy of dimethyl fumarate similar to the adult population, demonstrating that pediatric patients can benefit from this treatment (5). Natalizumab can be used in the case of very active diseases. Although not officially approved, various studies have shown excellent efficacy. Unfortunately, the risk of PML in children is not estimated due to the small samples that have been treated (40, 59). Clinical trials are currently underway on other highly effective drugs, including Ocrelizumab, Ofatumumab (anti-CD20) and Alemtuzumab (anti-CD52, LemKids trial) (52).

### 2.4. Engagement in POMS

In the management of POMS, it is essential an active involvement of the patient, the so-called “patient engagement,” and his caregivers, usually represented by the parents. This main goal has to be pursued from the very first visits and at the communication of the diagnosis (2, 67). The days after MS diagnosis represents a time of great stress for families, and an overload of information could create confusion, misunderstandings, or false expectations. Therefore it is necessary to provide adequate personalized advice, easily understandable and exhaustive, for example, iconographic stories. A better comprehension of the disease facilitates patient involvement (107). Due to the enormous impact of the disease, a multidisciplinary team composed of a neurologist, psychologist, and nurse who can meet the needs of patients and caregivers is essential. Adequate psychological support will be needed in dealing with the disruption of social life and family relationships. Therefore, it is important to encourage meetings between patients of the same age and between families of patients. Doctors must go beyond the simple medical care role by becoming a motivator. The young patient should feel part of a team that aims to fight the disease. The main weapons available are DMTs. In the past, injection therapy was the mainstay of treatment, with known and limited side effects. Otherwise, new, more effective, potentially dangerous oral drugs are now available. Therefore, the neurologist must debate the risk–benefit ratio with the patient and family members, encouraging their involvement in the decision-making process to choose the best treatment. Overcoming fear of therapy allows for avoiding harmful coping mechanisms in the future, encouraging better therapeutic adherence even in adulthood.

### 2.5. New communications tools: the digital

Our lifestyles and cognitive systems have changed throughout our evolutionary history, alongside human inventions, such as primitive tools, spoken language, writing, and arithmetics. Since the 1970s the Internet and the subsequent technological revolution have led to profound transformations of the human mind, thoughts and our way of life. In recent years the technological revolution has predictably reached the medical field as well, for example, in the field of cardiology, with FDA-approved devices to detect cardiac arrhythmias (95). Moreover, the current COVID-19 pandemic has further accelerated the technological transition in medicine and the role of telemedicine (78). Digital innovation is also emerging to monitor disease courses among patients with MS. A recent review (31) showed that digital technology has become part of clinical trials and was used to provide psychotherapy and motor rehabilitation with exergames, e-training, and robot-assisted exercises. Digital technology is particularly useful to standardize previously existing outcome measures, with automated acquisitions, reduced inconsistencies, and improved symptom detection (e.g., electronic recording of motor performance). Other clinical trials have used digital technology to monitor otherwise difficult-to-detect symptoms (e.g., fatigue, balance), to measure treatment adherence and side effects, and for self-assessment purposes. The collection of outcome measures is gradually shifting from on-site paper collection to Internet-based and, in the future, home-based Internet-based collection, with the detection of clinical and treatment characteristics that would otherwise have remained invisible. Similarly, remote interventions offer new possibilities for motor and cognitive rehabilitation. The role of technology in the therapeutic armamentarium to support MS patients appears to be of greater interest to pediatric patients, as they are more familiar than older patients with this latter. Examples included a recent trial that demonstrated the efficacy of an app in reducing stress and anxiety in POMS (22). Moreover, several studies explored the usefulness of digital technologies in POMS for motor exercise training program (114), physical activity and cognitive interventions (i.e., social-cognitive theory based) (70, 105).

### 2.6. Unmet needs: fears and confusions of POMS

POMS can be considered an “orphan” disease to all intents and purposes. Several studies have shown that there are still many unmet needs reported by children and adolescents affected by POMS, resulting not only from the physical and psychological effects of the disease in this specific age group but also from a lower availability of diagnostic and therapeutic information compared to the adult population (39). A recent meta-analysis analyzed 26 studies, including over 2,000 patients with POMS highlighting a profound negative impact on domains such as school performance, sociability and physical performance (39). Specifically, the lack of adequate knowledge of the disease has been reported as one of the main barriers experienced by patients with POMS in carrying out their daily activities, with important repercussions on the possibility of social integration. Several innovative ways have been proposed to improve communication between neurologists and caregivers to counter the sense of isolation that children and adolescents with MS often experience concerning the incorrect perception of being “different” from their peers due to illness. POMS patients, healthcare professionals and family members must adopt open communication, providing information in an age-appropriate and simple way. Taking the time to clearly explain the procedures, upcoming tests and treatments is essential to develop a comprehensive knowledge of MS, minimizing the risk of feeling confused and scared. From this point of view, modern technologies (i.e., telemedicine, online support groups) can represent a tool for sharing one’s experiences and clarifying doubts and perplexities about symptoms, fears and personal expectations (65). Furthermore, constant psychological support, also aimed at family members, can reduce the negative impact of the pathology on the quality of life of patients with POMS and redefine a new balance in family relationships, especially in the months next to the diagnosis.

## 3. Late-onset multiple sclerosis patients

### 3.1. Genetic background and environmental factors

LOMS and VLOMS patients remain relatively under-investigated in the literature (92). The same risk factors for the adult MS population are probably responsible for the late forms. The duration and the onset of exposure to environmental factors could possibly influence the age at onset in MS patients (i.e., air pollution with exposure to PM10, PM2.5, and O3 or cigarette smoke later in life; Figure 1) (55, 57). However, these assumptions need further confirmation. MS is more frequent in adult females than males, but this gender difference appears less marked in LOMS patients (56), possibly related to hormonal variations. A study by Baroncini et al. highlighted a greater risk of disability progression after menopause (14), probably concerning age-related neurodegeneration phenomena (19). Since patients with LOMS have a greater chance of presenting a progressive phenotype and a lower relapse rate (30), it is possible to hypothesize that hormonal variation may influence the age at onset. However, specific literature in this regard is not currently available. Furthermore, a greater relapse risk for women in the puerperium is known in the literature; a reduced birth rate could also be associated with a “delay” in time to the first clinical episode, thus postponing the clinical onset of the disease (57, 58). Some studies would then have indicated an increased risk of MS in men with low testosterone levels (117). Although age-related hormonal variations in men are not precocious and stereotyped, immunosenescence and hormonal factors may explain late onset in the male population. This area certainly deserves further investigation in the future. In contrast, the type of premorbid diet and the subsequent risk of developing LOMS was not shown to be associated in a large Danish registry study (92). Elderly patients also present numerous comorbidities more typical of the elderly subject, such as hypertension, dyslipidemia, and atherosclerotic processes with phenomena of chronic inflammation and oxidative stress, which could further influence the later onset of MS (64). The presence of such comorbidities might delay MS diagnosis in elderly patients; plus, evidence suggest that patients with vascular comorbidities will experience a faster progression. Finally, from a genetic point of view, while various studies have highlighted an earlier age of onset in association with several HLA alleles (for example, carriers of the HLA-DRB1*15 allele develop the disease earlier than non-carriers), few data are instead present for the patient LOMS and VLOMS (104). In an Australian study, the HLA-DRB1 *0801 allele was overrepresented in patients with LOMS, indicating a possible different genetic substrate even in late-onset patients (94).

### 3.2. Immunosenescence

Aging is a physiological process, typically occurring with the passing of the years, characterized by the progressive decline of bodily biological functions. This process is a major contributor to several comorbidities that frequently arise in the elderly (33). When this functional decline concerns the immune system, it is called immunosenescence. Modified proliferation and maturation of immune cells characterize immunosenescence and hempen the ability to develop an appropriate immune response. This leads to increased susceptibility to infection, improved autoimmune processes, and a worse response to vaccination (33). On the other hand, a compromised immune system causes a chronic inflammatory state, called “inflamm-aging,” with an increased level of inflammatory cytokines, which increases the risk of morbidity in the elderly population (12, 96). Chronic infections, such as Epstein–Barr virus (EBV), and accumulated senescent cells are responsible for inflammation and increase inflammatory cytokines, growth factors, and autoreactive antibody levels.

The age-related changes in the immune system mainly involve the adaptive immune system compared to the innate immune system. Indeed, a lower number of T, NK, and B naïve cells and an altered balance between pro- and anti-inflammatory molecules are typical features of immune system aging (33). Moreover, telomerase activity is generally reduced, leading to cellular senescence, interrupted proliferation, and a higher level of cell death (96). On the other hand, less effective phagocytosis, degranulation, and production of reactive oxygen species (ROS) characterize the senescent innate immune system and are responsible for higher susceptibility to viral and bacterial infections.

#### 3.2.1. Immunosenescence and MS

MS etiology is not fully known (80). Autoreactive CD4+ T lymphocytes, crossing the blood–brain barrier (BBB), infiltrating the CNS, and recognizing myelin antigens as not-self seems one of the pathogenetic determinants of MS. This process activates microglia and astrocytes, induces oligodendrocytes’ apoptosis, and leads to demyelination and axonal loss (37). All MS patients, including POMS, are characterized by an early immunosenescence since disease onset, with shorter telomerase, thymic dysfunction, increased CD4+/CD28- T Lymphocytes and memory T cells levels, reduced number of naïve T cells, and less functional regulatory T cells (9). The immune system’s premature senescence seems essential for the onset of MS and its progression (33). The evolution of the disease towards progressive forms and the progression independent of relapse activity (PIRA) is more tightly associated with immunosenescence and early neurodegeneration than with disease duration and patients’ age (33). Moreover, sex has been reported to influence immunosenescence based on genetic, epigenetic, lifestyle, environmental, and social differences (19). For instance, the adaptive immune system tends to reduce its efficacy earlier in men (51). Hormonal changes are also important in MS progression (i.e., protective role in the third trimester of pregnancy and higher relapse risk in postpartum), as in immunosenescence. Estrogens, in fact, show neuroprotective effects in animal models of autoimmune encephalitis, binding beta receptors, activating oligodendrocytes, macrophages, and dendritic cells, and supporting remyelination and recovery from axonal loss (14). Thus, reduced estrogens level in menopause leads to reproductive, neurological, and immunological changes in MS women in the direction of a worsening of disease and disability (19).

#### 3.2.2. Immunosenescence and elderly MS population

In the last decades, the number of LOMS and VLOMS has been growing, in line with the prevalence of elderly MS patients (17). Reduced cerebral plasticity and growth factor levels are typical of this population and lead to incomplete recovery from demyelination and diffuse axonal degeneration. Moreover, in the aging BBB, permeability increases, leading to a higher degree of inflammatory cells infiltrating CNS and facilitating astrocyte proliferation and glial scars development. This phenomenon contributes to incomplete recovery from demyelination and myelin debris clearance. The prevalence of progressive forms of MS is higher with increasing age (110). A higher number of B memory and plasma cells are typical of these forms and form lymphatic follicles. Increased numbers of memory B cells and plasma cells are characteristic of these forms and organize into meningeal ectopic follicles. In addition, with the transition to progressive MS, BBB permeability gradually decreases, leading to the compartmentalization of disease activity and significantly dampening therapeutic efficacy. Oxidative processes also increase and phagocytosis becomes much less efficient with aging. These mechanisms are responsible for progressive iron accumulation in the brain and in active chronic lesions called “smoldering lesions” (1, 62). These slowly expanding lesions are typical of elderly patients and are more frequent in long-term disease (1). Histologically, these are characterized by a central astrocyte scar and a peripheral rim of active macrophages full of iron and increased oxidative processes. Other radiological features of LOMS are global and regional cerebral atrophy (typical of grey matter), white matter lesion load atrophy (especially of periventricular lesions, responsible for the accumulation of cerebrospinal fluid) and increased cortical lesions number (connected to higher cognitive disability) (20).

In summary, immunosenescence results in such biological and immune changes in LOMS that it can be considered a major determinant of increased disability in this population.

### 3.3. Treatments in LOMS and elderly patients: safety, discontinuation, and engagement

There is a lack of data on the safety and efficiency of DMTs in older people with MS (112). Patients over 55 are usually excluded from clinical trials (112). Therefore, it is very difficult to determine whether the treatments available for older people are safe and effective. Consequently, the decision to initiate DMTs in elderly patients should be carefully considered, considering the risks associated with the therapy and its limited efficacy. An Italian study on natalizumab-related Progressive Multifocal Leukoencephalopathy (PML) showed that older age at natalizumab (NTZ) start might be a risk factor for developing PML before 24 infusions (93). PML risk is probably related to a major susceptibility secondary to the immunosenescence process.

Regarding other DMTs, Fingolimod has recently been associated with cases of PML, and these also seem to have an age-dependent trend (45). Furthermore, PML in elderly patients appears to have a worse outcome in various studies: in fact, most fatal cases of PML are in elderly patients (99). Age-associated changes in humoral immunity reduce the ability to mount an effective antibody response, suggesting that age may represent an additional stratified risk for PML in patients treated with MS therapy (45). Higher age is also a risk factor for other types of infections: for example, a higher risk of VZV reactivation was seen in older patients receiving Fingolimod, Cladribine, Natalizumab, and Alemtuzumab (47). Cryptococcal meningitis also appears to have the same age-related trend in patients receiving Fingolimod (115).

Nevertheless, due to the wide clinical variation in this group of patients, it is essential to individualize the treatment. Schweitzer et al. (99) suggested that the benefits of high-efficacy DMTs could decrease with age. Weideman et al. (115, 116) also found that the efficacy of DMTs was negatively correlated with age. In another work, age over 53 predicted no efficacy of DMTs (88). Older patients present significant pharmacokinetic and pharmacodynamic differences compared to younger patients.

Moreover, in advanced ages, progressive forms are more prevalent than RRMS. Despite expanding the therapeutic arsenal in MS, only one drug is approved for treating the primary progressive forms (Ocrelizumab). This drug showed a 24% reduction in the risk of disability progression compared to the placebo (36). Mitoxantrone and Siponimod have shown positive results in secondary progressive forms (53, 72, 118), although evidence suggests that the benefit is most evident in patients with persistent inflammatory activity (72).

Another important factor to consider before starting a DMT is the risk of developing cancer in ancient people: actually, most of the studies on the current DMTs have not shown a real correlation between their use and a greater risk of developing tumors; however, due to the increased cancer risks in individuals of advancing age, the interactions of age, immunosenescence, and DMTs use needs further study (99).

An Italian study on naïve RR LOMS patients did not show statistical differences between injective and oral treatment regarding time to the first relapse, risk of disability, and treatment withdrawal (118). Another problem to discuss is the treatment discontinuation in light of the decreased efficiency and increased risk of DMT in elderly patients. Another important factor to consider in this type of patient is the treatment discontinuation problem. A recent study (53) highlighted how discontinuation of treatment in elderly and previously stable patients results in possible new worsening/progression of the disease. These findings are very interesting, considering the growing number of older patients with MS in recent years and the many uncertainties about how to treat them. As previously reported, it’s generally accepted that older patients will benefit less from currently available DMTs. However, most studies involved ancient therapies; today, DMTs with better efficacy and safety profiles are available. The study, which involved adult patients of all ages, also found that disease worsening and progression resulting from therapy discontinuation were independent of patient age. The type of MS (RR versus progressive) also did not seem to influence disease progression. Notably, up to 40% of previously stable progressive patients showed worsening in disability after drug discontinuation. Recently, the ongoing DISCO-MS study evaluated discontinuation of DMT in participants aged 55 years or older, clinically stable (no relapses) for at least 5 years, and radiologically stable for three or more years. The study is probably the largest controlled study for DMTs conducted in MS patients older than 55. There is not a significant difference between treated and untreated patients regarding clinical activity and clinical worsening. Clinical relapses were particularly rare. An increase in the number of MRI lesions was highlighted in the group that stopped therapy: however, MRI changes involved a reduced number of new lesions (1 or 2 lesions), and numerous observational studies have shown that 1–2 new brain lesions on MRI scan after 1 year of therapy, are not associated with a significant risk of disability progression in the following 5–10 years. On the other hand, a higher number of lesions (3 or more new lesions), an active lesion or new relapses appear to correlate with significant disability progression. Thus, minimal evidence of new disease activity may be functionally acceptable in elderly patients after DMTs suspension. Therapy discontinuation is also a minor risk factor for elderly patients (over 55 years) with moderate disabilities. Interruption of therapy should be considered in patients with secondary progressive (SP) disease due to the poor therapeutic efficacy of most drugs in this disease stage. A retrospective study in patients with SPMS demonstrated that after discontinuation of INFβ or glatiramer acetate therapy, the rate of relapse and progression of disability remained similar to treated patients (18). In this context, it is now clear how it is essential to maintain a direct and one-to-one relationship between patient and neurologist in the therapeutic management of a chronic and complex pathology such as multiple sclerosis. It is known that older adults with MS are more likely to have a reduced health-related quality of life as a consequence of increased social isolation, the development of cognitive impairment, which, together with a physical disability, multiple comorbidities, and therefore to polytherapy, lead to a greater sense of dependence and “uselessness” (21).

Based on these considerations, an increasing number of Patient Health Engagement projects is emerging to guarantee shared treatment management (drug start and stop) and socioeconomic and work aspects. One of the main challenges of the last years is increasing the patient’s awareness of being a central point in the process of therapeutic decision. The active involvement of the MS patient should occur at any age through educational, listening, and empowerment programs. The purpose of these programs is to allow greater trust between doctor and patient and, consequently, greater autonomy and proactivity of the patient in the management of own lifestyle, health and care (97). The availability of new drugs, with their advantages and disadvantages, necessarily requires sharing the therapeutic choice, whether to start, continue or discontinue a drug, based on the single patient and needs. Several experts have reported how patients of MS appreciate direct and sincere communication, even when medical data are uncertain, resulting in better satisfaction of healthcare and so, greater adherence to the treatment (49).

Finally, in the elderly patient, it is essential to start a concomitant process of active involvement of caregivers (Caregiver Engagement), who play a fundamental and complementary role in the therapeutic, rehabilitative and social management of these patients.

## 4. Final remarks and conclusion

POMS and LOMS are two demographic extremes with different pathogenesis, clinical management, therapeutic approach, and social engagement. Both forms are the result of a complex gene–environment interaction, whereby the disease would emerge from a condition of genetic susceptibility under the impulse of one or more factors of environmental exposure. In this regard, both in POMS and above all in LOMS, immunosenescence could play an important role. There are many therapeutic challenges in these categories of patients and there is a lack of data on the safety and efficiency of DMTs especially in LOMS. However early DMTs start is strongly recommended and a multidisciplinary team that can meet the individual needs of the patient and caregivers is essential. A current challenge is the role of digital innovation in supporting the patient, especially POMS, not only in psychological and rehabilitation monitoring and support, but also in improving communication between the neurologist and the patient/family members, in a process of active involvement of patients and caregivers.

## Author contributions

RL and MC contributed to conception, design of the study, and contributed in writing—review and editing the original draft. NC and EV organized the search literature. AC, BG, MF, EP, AN, FM, MN, GS, SS, NC, and EV wrote the first draft of the manuscript. All authors contributed to the article and approved the submitted version.

## Funding

This work was supported by Merck Serono S.p.A., Rome, Italy, an affiliate of Merck (CrossRef Funder ID: 10.13039/100009945). Merck had no influence in the design, interpretation of the data, or content of the manuscript. No payments were made to the authors for the writing of this manuscript. Editorial assistance was provided by Ethos, and supported by an independent medical writing grant from Merck Serono S.p.A., Rome, Italy.

## Conflict of interest

RL and MC received financial compensation for attendance to expert meetings as part of an educational programme by Merck Serono S.p.A., Rome, Italy, an affiliate of Merck.

The remaining authors declare that the research was conducted in the absence of any commercial or financial relationships that could be construed as a potential conflict of interest.

## Publisher’s note

All claims expressed in this article are solely those of the authors and do not necessarily represent those of their affiliated organizations, or those of the publisher, the editors and the reviewers. Any product that may be evaluated in this article, or claim that may be made by its manufacturer, is not guaranteed or endorsed by the publisher.
